# Analysis and Design of SCMA-Based Hybrid Unicast-Multicast Relay-Assisted Networks

**DOI:** 10.3390/s19020329

**Published:** 2019-01-15

**Authors:** Yibo Zhang, Xiaoxiang Wang, Dongyu Wang, Yufang Zhang, Yanwen Lan

**Affiliations:** Key Lab. of Universal Wireless Comm., Ministry of Education, Beijing University of Posts and Telecommunications, Beijing 100876, China; yibo@bupt.edu.cn (Y.Z.); dy_wang@bupt.edu.cn (D.W.); yf910309@bupt.edu.cn (Y.Z.); yanwen@bupt.edu.cn (Y.L.)

**Keywords:** SCMA, wireless multicast, relay, cooperative transmission, outage probability, optimal relay design

## Abstract

This paper studies a multi-user network model based on sparse code multiple access (SCMA), where both unicast and multicast services are considered. In the direct transmission scheme, the communication between the base station (BS) and the users is completed with one stage, in which the relay is inexistent. In the two-stage cooperative transmission scheme, any number of relays are placed to improve the reliability of wireless communication system. The BS broadcasts the requested message to users and relays in the first stage, and the successful relays forward the message to unsuccessful users in the second stage. To characterize the performance of these two schemes, we derive the exact and approximate expressions of average outage probability. Furthermore, to take full advantage of the cooperative diversity, an optimal power allocation and relay location strategy in the high signal-to-noise ratio (SNR) regime is studied. The outage probability reaches the minimum value when the first stage occupies half of the total energy consumed. Simulation and analysis results are presented to demonstrate the performance of these two schemes. The results show that the two-stage cooperative scheme effectively reduce the average outage probability in SCMA network, especially in the high SNR region.

## 1. Introduction

As an efficient method of physical resource management, non-orthogonal multiple access (NOMA) is considered as a promising technology to achieve the targets of the fifth generation (5G) wireless communication systems (e.g., high capacity, massive connectivity, and low latency) [1]. Among the available NOMA technologies, sparse code multiple access (SCMA) [2] is a code domain multiplexing scheme, which can achieve an obvious increase in spectral efficiency compared to orthogonal multiple access (OMA). Different from low-density signature [3], in SCMA, the incoming bits are mapped to sparse codewords selected from the predefined codebook [4]. The SCMA encoder combines bits modulation and spreading together, and shaping gain is obtained from the multi-dimensional constellation [5]. Multiple access is achieved by assigning different codebooks to different users, the codebook consists of some parameters of the bit modulation, such as spectrum resource, power, and multi-dimensional constellation points. Since the same time-frequency resource block can be shared by different codebooks, SCMA can obtain overloading gain. As a result of overload, the multi-user detection (MUD) technology is required. Benefiting from the sparsity feature of the codeword, message passing algorithm (MPA) can get a near optimal performance with low complexity [6].

Similar to NOMA, wireless multicast is one of the technologies that is most likely to be applied to 5G and beyond [7,8], with the purpose of saving physical resources and satisfying future point to multipoint services. In the multicast system, the base station (BS) serves multiple users with a single physical resource because these receivers require the same content. To improve the performance of the multicast systems, two-stage cooperative transmission scheme has attracted wide attention [9,10,11,12,13]. For example, in [9], the outage probability and resource allocation for the cooperative multicast scheme are studied. Ref. [10] proposed a probability-based power control method in cooperative multicast communications to minimize the user power consumption. Ref. [11] investigated the energy efficient cooperative multicast, aiming to minimize the total transmission power.

There is an important observation that both NOMA and wireless multicast can alleviate the shortage of spectrum resource in 5G networks. Therefore, it is meaningful to study the performance of NOMA-based multicast systems. Most recently, Ref. [14] designed a novel cooperation strategy for NOMA unicast-multicast, in which a multicast user is selected to forward a superposed message to unsuccessfully decoded unicast and/or multicast users. Ref. [15] did research on the use of NOMA for mmWave cooperative multicast system. In [16], the application of NOMA to multicast cognitive radio networks is investigated, and a dynamic cooperative transmission scheme is proposed. However, these studies are all focused on the power domain NOMA, and their results are not suitable for the SCMA system.

The research for the SCMA system is mainly focused on codebook design [4,17,18], MUD technology [19,20,21], and performance evaluation [22,23,24,25]. For example, Ref. [4] applied lattice rotation to codebook design and proposed a systematic approach. In [17], joint codebook design and assignment are considered to minimize the algorithm complexity of MPA. In order to take full advantage of the sparse characteristic of SCMA codebook, the codebook design and the advanced decoding strategy are studied in [18]. Ref. [19] investigated the improved MPA detector for SCMA, which could get a better error performance and a higher convergence speed. The performance evaluation of SCMA is studied in [22], in which a linear sparse sequence modeling is used to derive the capacity of downlink multi-user SCMA system. Ref. [23] applied SCMA into device-to-device (D2D) communication system and investigated the performance of SCMA in D2D and cellular hybrid network.

However, all the aforementioned research efforts on SCMA are limited in wireless unicast, where the message transmitted by the BS can only be received by one user. In these SCMA unicast strategies, if some users have the common interest (e.g., live sports streaming, video conferencing, etc.), the same data has to be sent separately, leading to low spectral efficiency and link utilization. Accordingly, when some users have the same interest, integrating cooperative multicast into SCMA can combine their advantages, and then improve the system capacity/reliability. Nevertheless, there is still a lack of the study on SCMA-based cooperative multicast systems.

For that reason, we propose a hybrid unicast-multicast downlink network, where SCMA is applied to unicast and multicast transmission. We divide users into two categories: the users who request the same content within the coverage of the BS, are called multicast users, and the BS transmits the message to these users using the same codebook simultaneously; the users who do not belong to a multicast group, are named unicast users, and the BS transmits the message to these users using different codebooks. The outage probability is used to measure the performance of the proposed network model, due to the diversity of channel gains for different receivers in the multicast group. To further improve the reliability of the unicast-multicast system, relay-assisted two-stage cooperative transmission scheme is introduced. The motivation for the proposed model is to take the advantage of SCMA and cooperative multicast to improve the spectral efficiency.

In this paper, a direct transmission scheme and two-stage cooperative transmission scheme in SCMA-based unicast-multicast hybrid network are developed. In the direct transmission scheme, the whole communication process is completed in one stage, where the relay is non-existent in the cell. In the two-stage cooperative transmission scheme, any number of relays are placed in the cell, and these relays are used to decode-and-forward (DF) the message to users. The BS broadcasts the requested message to users and relays in the first stage, and failed users receive the messages from multiple successful relays using a maximal ratio combiner (MRC) in the second stage. For the two-stage cooperative transmission scheme, the optimal relay design is considered to improve the performance of the system. Considering a delay constraint network, the BS transmits the message with a target rate for each unicast user/multicast group, the average outage probability is used to indicate the performance of the proposed network model. The main contributions of this work are summarized as follows:

- We propose a network model of hybrid unicast-multicast based on SCMA, in which two transmission schemes (direct transmission and relay-assisted two-stage cooperative transmission) are discussed. The proposed strategy is able to achieve high spectral efficiency and link utilization.
- Based on the presented network model, we derive the expressions of outage probability for these two schemes. From the results of calculation and simulation, we can observe that SCMA outperforms OMA in terms of average outage probability. We can also observe that the outage performance of cooperative transmission is better than direct transmission.
- For the two-stage cooperative transmission scheme, relay design is considered to improve the system performance. We first obtain an asymptotic approximation of average outage probability in the high signal-to-noise ratio (SNR) regime. Then, based on this tight asymptote, we are able to determine the optimal relay locations and power allocation for the cooperative scheme. Simulation results show that the proposed system design scheme can effectively reduce the average outage probability.

The rest of this paper is organized as follows: Section 2 presents the system model of direct transmission and cooperative transmission. In Section 3, the outage probabilities of two transmission schemes are analyzed, and their expressions are derived, respectively. Approximation of outage probability is studied in Section 4, and the optimal relay location and power allocation are obtained. In Section 5, the simulation results of two transmission schemes are demonstrated and discussed. The conclusions of this work are provided in Section 6.

## 2. System Model

### 2.1. Network Model

**Figure 1:**
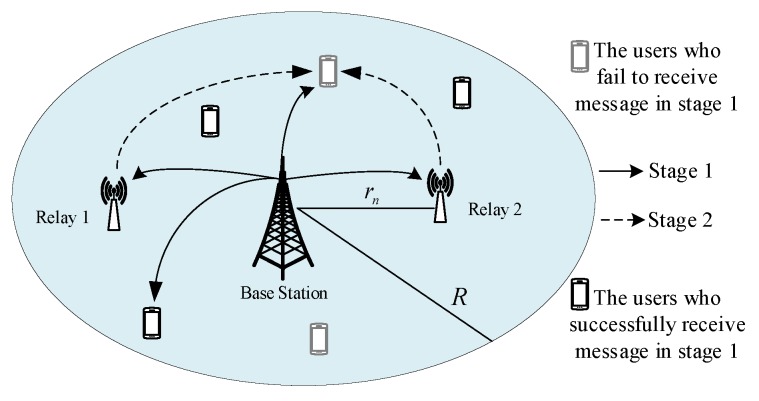
Network model.

We consider a downlink single-cell network as shown in Figure 1, where a BS at the center of the cell with a radius of *R*. The users, denoted by U={1,2,..u,..U}, are uniformly distributed in the cell. The BS allocates the same physical resources to the users who request the same content, and these users form a multicast group. The BS allocates different physical resources to users requesting different content. Assuming that the locations of different users are independently and identically distributed (i.i.d.), the distance between user *u* and BS is $r_{u}$, and the angle is $\theta_{u}$. Then, the probability densities of $r_{u}$ and $\theta_{u}$ are $\frac{2\pi r_{u}}{\pi R^{2}}$ and $\frac{1}{2\pi }$, respectively. Accordingly, the marginal distribution of $r_{u}$ and joint probability density function of ( $r_{u}$, $\theta_{u}$) can be expressed as

(1) $$\begin{array}{c} \left\{\begin{array}{c} f\left(r_{u}\right)=\frac{2r_{u}}{R^{2}},0\leq r_{u}\leq R.\\ f\left(r_{u},\theta_{u}\right)=\frac{r_{u}}{\pi R^{2}},0\leq r_{u}\leq R,0\leq \theta_{u}\leq 2\pi . \end{array}\right. \end{array}$$

Following the work in [9], we deploy $N_{s}$ relays in the cell to help DF the message, which can be placed at optimal locations to minimize the outage probability. Let $N_{s}=\;\;\left\{\;\;\;1,2\ldots n\ldots N_{s}\right\}$ be the set of all relays, and $\left(r_{n},\left(n-1\right)2\pi /N_{s}\right)$ be the location of relay *n*. In this work, we assume that all devices are equipped with a single antenna and work in half-duplex mode, and the channel state information (CSI) is obtained perfectly. The channel gain between any two nodes is subject to independent narrow-band Rayleigh fading *h*, path loss exponent η(η>2), and additive white Gaussian noise with zero mean and variance $N_{0}$, where h∼CN(0,1) is modeled as a zero-mean circularly symmetric complex Gaussian random variable with unit variance.

### 2.2. Sparse Code Multiple Access

**Figure 2:**
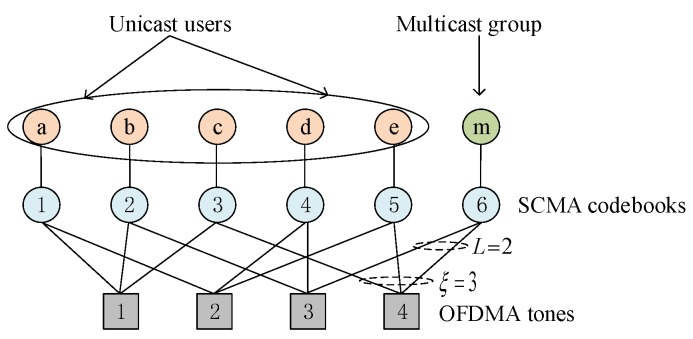
An exemplary mapping between SCMA codebooks and OFDMA tones.

In an SCMA encoder, each user’s data stream with the length of $\log_{2}M$ bits is directly mapped to a *K*-dimensional complex codeword selected from a codebook, where *M* is the codebook size, *K*-dimensional codeword over *K* orthogonal resources (e.g., orthogonal frequency-division multiple access (OFDMA) tones). For each codeword, it is a sparse vector with L(L<K) non-zero elements. Each SCMA layer is assigned with a codebook, and an example of the mapping relationship between SCMA layers and OFDMA tones is shown in Figure 2. Let C={1,..,c,…,C} represents the codebook set of an SCMA block, where the number of codebooks C(C>K) is a function of the codeword length *K* and the number of non-zero elements *L*. This work considers the regular codebook, then, the number of non-zero elements in each codebook is equal to *L*. In the SCMA network, the resource unit is codebook instead of OFDMA tone, and multiple access is achieved by assigning different codebooks to different users. Since multiple codebooks on an SCMA block share the same time-frequency resources, SCMA can achieve overload gain compared to OMA.

To correctly demodulate the data of each layer, the superimposed signal must be separated at the receiver. Benefitting from the sparsity of SCMA codewords, there are only partial (ξ) codebooks colliding at a tone (e.g., ξ=3 for each tone in Figure 2). Therefore, the SCMA receivers utilizing MPA get a near optimal performance with low complexity. In this study, the case of codebook reuse is not considered, and there is no mutual interference among links.

### 2.3. Direct Transmission

We consider that an SCMA block contains *C* codebooks, and each unicast user/multicast group occupies one codebook. All users in a multicast group are assigned the same codebook because they request the same content. Let *G* be the number of multicast groups, *S* be the number of unicast users, G+S=C, and the set of all users is U. The power of each codebook is assumed to be equal and is equally distributed among *L* codewords as described in [22]. Assuming the total transmit power of each tone in direct transmission scheme is *P* and duration is *T* accordingly, thus, the power of each codeword is $P_{d}=P/\xi$. The received signal of user u∈U is a superposition of *C* *K*-dimensional vectors, which can be expressed as

(2) $$\begin{array}{c} y_{u}^{d}=\sum_{c\in C}\mathrm{diag}\left(h_{u}\right)\sqrt{P_{d}{r_{u}}^{-\eta }}x_{c}+n_{u}, \end{array}$$

where $h_{u}=\left(h_{u,1},\ldots ,h_{u,k},\ldots ,h_{u,K}\right)$ is channel vector, the form diag(·) represents the diagonal matrix, $h_{u,k}$ represents the Rayleigh fading between the BS and the user *u* over *k*-th OFDMA tone, $r_{u}$ is the distance from BS to user *u*, $x_{c}$ is the selected *K*-dimensional symbol from codebook *c*, and $n_{u}$ is the noise vector of user *u*. Here, the subscript “u” is user index and “d” represents the direct transmission scheme.

Without loss of generality, the transmission rate of each codebook is assumed to be equal and represented by $I_{f}$. Therefore, given $I_{f}$ and $r_{u}$, the outage probability of user *u* in direct transmission scheme when using codebook *c* can be expressed as [25,26,27]

(3) $$\begin{array}{c} P_{u,c}^{d}=\mathrm{Pr}\left(\gamma_{u,c}^{d}<2^{I_{f}}-1\right), \end{array}$$

where Pr(·) represents the probability of the inner event, $\gamma_{u,c}^{d}=\sum_{k\in K_{c}}P_{d}{|h_{u,k}|}^{2}{r_{u}}^{-\eta }/N_{0}$ is the SNR of user *u* on codebook *c*, and $\left|\right|K_{c}\left|\right|=L$ is the set of non-zero OFDMA tones occupied by codebook *c*.

### 2.4. Two-Stage Cooperative Transmission

In the cooperative transmission scheme, any number of relays at fixed positions are placed to help forward packets. The whole communication process is divided into two stages, and the duration of each stage is T/2. In the first stage, the BS multicasts the requested message to all users and relays. There are two states (successful or failed to demodulate the message) of these users and relays, and the information of these two states will be fed back to the BS. The second stage is necessary if there are failed users. In the second stage, the BS resends the message when all the relays failed. Otherwise, successful relays forward the message to failed users simultaneously, and failed users use MRC to combine the signals from these relays. The specific process of two-stage cooperative transmission scheme is described in Algorithm 1.

**Algorithm 1** Two stages cooperative transmission scheme.
1:
**The first stage:**
2:The BS multicasts the requested message to all users and relays.3:Calculate the demodulation results of each user and relay.4:
**The second stage:**
5:**if** All users successfully received. **then**6: Break (i.e., end of transmission).7:**else if** All relays failed. **then**8: The BS resends the message to the failed users.9:
**else**
10: All successful relays forward the message to failed users simultaneously.11: The failed users combine the signals from these relays using MRC.12:
**end if**
13:
**Transmission is completed.**



In the first stage, the received signal of user *u* can be written as

(4) $$\begin{array}{c} y_{{}_{u}}^{s1}=\sum_{c\in C}\mathrm{diag}\left(h_{u}\right)\sqrt{P_{s1}{r_{u}}^{-\eta }}x_{c}+n_{u}, \end{array}$$

where $P_{s1}$ is the transmit power of each codeword in stage one, “s1” means the first stage of cooperative transmission scheme, and the other variables are the same as Equation (2). As the whole process is divided into two stages, the outage rate threshold of cooperative transmission scheme is $2I_{f}$, then the outage probability of user *u* in the first stage when using codebook *c* is given by

(5) $$\begin{array}{c} P_{u,c}^{s1}=\mathrm{Pr}\left(\gamma_{u,c}^{s1}<2^{2I_{f}}-1\right), \end{array}$$

where $\gamma_{u,c}^{s1}=\sum_{k\in K_{c}}P_{d}{|h_{u,k}|}^{2}{r_{u}}^{-\eta }/N_{0}$ is the SNR of user *u*.

Different from the users, the relays have all *C* codebooks to help forward. For relay *n*, its received signal can be written as

(6) $$\begin{array}{c} y_{{}_{n}}^{s1}=\sum_{c\in C}diag\left(h_{n}\right)\sqrt{P_{s1}{r_{n}}^{-\eta }}x_{c}+n_{n}, \end{array}$$

where $h_{n}=\left(h_{n,1},\ldots ,h_{n,k},\ldots ,h_{n,K}\right)$ is the channel vector, $h_{n,k}$ represents the Rayleigh fading between the BS and the relay *n* over *k*-th OFDMA tone, $n_{n}$ is the noise vector, and the other variables are the same as (2). The outage probability of relay *n* on codebook *c* in stage one is given by

(7) $$\begin{array}{c} P_{n,c}^{s1}=\mathrm{Pr}\left(\gamma_{n,c}^{s1}<2^{2I_{f}}-1\right), \end{array}$$

where $\gamma_{{}_{n,c}}^{s1}=\sum_{k\in K_{c}}P_{s1}{|h_{n,k}|}^{2}{r_{n}}^{-\eta }/N_{0}$. Assuming *N* relays decode the message of codebook *c* correctly in stage one, and the set of these relays is denoted by $N=(n_{1},\ldots n_{i},\ldots ,n_{N})$. Then, the probability that relays in N decode the message of codebook *c* correctly in the first stage is given by

(8) $$\begin{array}{c} P_{N,c}=\prod_{n_{i}\in N}\left(1-P_{n,c}^{s1}\right)\times \prod_{n_{i}\notin N}P_{n,c}^{s1}. \end{array}$$

In the second stage, there are two cases of N≥1 and N=0. In the case of N≥1, at least one relay demodulates the message successfully, and these relays forward the message to the failed user. Users who failed in the first stage use MRC to combine the signals from all successful relays. Accordingly, the received signal of user *u* in stage two can be expressed as

(9) $$\begin{array}{c} y_{u}^{s2^{†}}=\sum_{n_{i}\in N}\sum_{c\in C}diag\left(h_{u,n_{i}}\right)\sqrt{P_{s2^{†}}{r_{u,n_{i}}}^{-\eta }}x_{c}+n_{u}=\sum_{c\in C}diag\left(h_{u,n_{i}}^{†}\right)\sqrt{P_{s2^{†}}}x_{c}+n_{u}, \end{array}$$

where “$s2^{†}$” represents the second stage of cooperative transmission scheme in the case of N≥1, $h_{u,n_{i}}=\left(h_{u,n_{i},1},\ldots ,h_{u,n_{i},k},\ldots h_{u,n_{i},K}\right)$, $h_{u,n_{i},k}$ represents the Rayleigh fading between relay $n_{i}$ and user *u* over *k*-th OFDMA tone, $r_{u,n_{i}}=\sqrt{{r_{u}}^{2}+{r_{n}}^{2}-2r_{u}r_{n}\cos\left(\theta_{u}-\left(n_{i}-1\right)2\pi /N_{s}\right)}$ is the distance between user *u* and relay $n_{i}$, $P_{s2^{†}}$ is the transmit power of each codeword on each relay in the second stage, and $h_{u,n_{i}}^{†}\overset{\Delta }{=}\;\sum_{n_{i}\in N}h_{u,n_{i}}\sqrt{{r_{u,n_{i}}}^{-\eta }}$. Since $h_{u,n_{i},k}∼CN\left(0,1\right)$, we have $h_{u,n_{i}}^{†}∼CN\left(0,\sum_{n_{i}\in N}{r_{u,n_{i}}}^{-\eta }\right)$. It is worth noting that the users obtain an array gain to improve the reliability of communication when N>1. Given $r_{u}$, $\theta_{u}$ and N, the outage probability of user *u* when using codebook *c* in the case of N≥1 is expressed as (Here, we assume that the user has discarded the signal received in stage one. These assumptions are widely used in the two-stage cooperative communication, e.g., in [28,29].)

(10) $$\begin{array}{c} P_{u,c}^{s2^{†}}=\mathrm{Pr}\left(\gamma_{u,c}^{s2^{†}}<2^{2I_{f}}-1\right), \end{array}$$

where $\gamma_{u,c}^{s2^{†}}=\sum_{k\in K_{c}}|h_{u,n_{i}}^{†}{|}^{2}P_{s2^{†}}/N_{0}$.

In the case of N=0, there is no relay which decodes the message correctly in stage one. In this situation, the BS resends the messages again, and all of relays do not work in the second stage. Then, the received signal of user *u* can be expressed as

(11) $$\begin{array}{c} y_{u}^{s2^{*}}=\sum_{c\in C}diag\left(h_{u}\right)\sqrt{P_{s2^{*}}{r_{u}}^{-\eta }}x_{c}+n_{u}, \end{array}$$

and the outage probability of user *u* when using codebook *c* is

(12) $$\begin{array}{c} P_{u,c}^{s2^{*}}=\mathrm{Pr}\left(\gamma_{u,c}^{s2^{*}}<2^{2I_{f}}-1\right). \end{array}$$

In Equations (11) and (12), “$s2^{*}$” represents the second stage of cooperative transmission scheme in the case of N=0, $\gamma_{{}_{u,c}}^{s2^{*}}=\sum_{k\in K_{c}}P_{s2^{*}}{\left|h_{u,k}\right|}^{2}{r_{u}}^{-\eta }/N_{0}$, and $P_{s2^{*}}$ is the transmit power of each codeword.

Considering Equations (10) and (12) together, the outage probability of user *u* in the second stage is given by

(13) $$\begin{array}{c} P_{u,c}^{s2}=\left\{\begin{array}{c} \mathrm{Pr}(\gamma_{u,c}^{s2^{†}}<2^{2I_{f}}-1),N\geq 1,\\ \mathrm{Pr}(\gamma_{u,c}^{s2^{*}}<2^{2I_{f}}-1),N=0. \end{array}\right. \end{array}$$

The outage of the user in cooperative scheme is defined as the user failed in both stages. According to Equations (7), (8), and (13), the outage probability of user *u* in cooperative transmission scheme when using codebook *c* can be expressed as

(14) $$\begin{array}{c} P_{u,c}^{s}=P_{u,c}^{s1}\sum_{N=0}^{N_{S}}\sum_{|N|=N}P_{N,c}P_{u,c}^{s2}, \end{array}$$

where the superscript “s” represents the cooperative transmission scheme.

To fairly compare the performance between direct transmission and cooperative transmission, we assume that the total energy consumed by the two schemes is equal. Hence, the transmission power of cooperative transmission scheme is subject to

(15) $$\begin{array}{c} PT=\left\{\begin{array}{c} \frac{T}{2}\xi \left(P_{s1}+NP_{s2^{†}}\right),N\geq 1,\\ \frac{T}{2}\xi \left(P_{s1}+P_{s2^{*}}\right),N=0. \end{array}\right. \end{array}$$

## 3. Average Outage Performance Analysis

In this section, the average outage performance of two transmission schemes is analyzed. We assume that the distance between the relay and the BS is fixed. The optimal locations of relays will be analyzed in the next section. Since h∼CN(0,1), if the distance between the transmitter and the receiver is given, the SNR of the receiver on each codebook can be expressed as the sum of *L* exponential distribution variables, that is subject to Gamma distribution. Then, we have

(16) $$\begin{array}{c} \left\{\begin{array}{c} \gamma_{u}^{d}∼\Gamma \left(L,\lambda_{u}^{d}\right)\\ \gamma_{u}^{s1}∼\Gamma \left(L,\lambda_{u}^{s1}\right)\\ \gamma_{n}^{s1}∼\Gamma \left(L,\lambda_{n}^{s1}\right)\\ \gamma_{u}^{s2^{†}}∼\Gamma \left(L,\lambda_{u}^{s2^{†}}\right)\\ \gamma_{u}^{s2^{*}}∼\Gamma \left(L,\lambda_{u}^{s2^{*}}\right), \end{array}\right. \end{array}$$

where

(17) $$\begin{array}{c} \left\{\begin{array}{c} \lambda_{u}^{d}=r_{u}^{\eta }N_{0}/P_{d}\\ \lambda_{u}^{s1}=r_{u}^{\eta }N_{0}/P_{s1}\\ \lambda_{n}^{s1}=r_{n}^{\eta }N_{0}/P_{s1}\\ \lambda_{u}^{s2^{†}}=N_{0}{\left(\sum_{n_{i}\in N}r_{u,n_{i}}^{-\eta }\right)}^{-1}/P_{s2^{†}}\\ \lambda_{u}^{s2^{*}}=r_{u}^{\eta }N_{0}/P_{s2^{*}}. \end{array}\right. \end{array}$$

In Equation (16), γ∼Γ(L,λ) represents the random variable γ subject to Gamma distribution, its shape parameter is equal to the number of non-zero elements *L*, and its rate parameter is λ. For random variable γ, its probability density function can be formulated as

(18) $$\begin{array}{c} f\left(\gamma ;L,\lambda \right)=\left\{\begin{array}{c} \gamma^{L-1}e^{-\lambda \gamma }\lambda^{L}/\left(L-1\right)!,\gamma >0,\\ 0,\gamma \leq 0, \end{array}\right. \end{array}$$

where (L−1)! represents the factorial of (L−1).

### 3.1. Average Outage Performance of Direct Transmission

According to Equations (16)–(18), we can get the probability density function of $\gamma_{u}^{d}$ is $f\left(\gamma_{u}^{d};L,\lambda_{u}^{d}\right)=\gamma {{}_{u}^{d}}^{L-1}e^{-\lambda_{u}^{d}\gamma_{u}^{d}}\lambda {{}_{u}^{d}}^{L}/\left(L-1\right)!,\gamma_{u}^{d}>0$. Using the Formula $\int_{t}^{\infty }x^{n}e^{-ax}dx=e^{-at}\sum_{i=0}^{n}\frac{n!t^{i}}{i!a^{n-i+1}},t>0,a>0,n=0,1,2,\ldots$ in [30], the conditional outage probability of user *u* in direct transmission scheme can be expressed as

(19) $$\begin{array}{c} P_{u}^{d}=\mathrm{Pr}\left(\gamma_{u}^{d}<2^{I_{f}}-1|r_{u}\right)=1-\mathrm{Pr}\left(\gamma_{u}^{d}\geq T_{d}|r_{u}\right)=1-\int_{T_{d}}^{\infty }f(\gamma_{u}^{d})d\gamma_{u}^{d}=1-\sum_{i=0}^{L-1}\frac{{\left(\lambda_{u}^{d}T_{d}\right)}^{i}}{i!}e^{-\lambda_{u}^{d}T_{d}}, \end{array}$$

where $T_{d}=2^{I_{f}}-1$. For *U* users with uniformly distributed in the cell, assuming all Rayleigh channel gains $\left\{h_{u}\right\}$ are i.i.d, the average outage probability of *U* users can be formulated as

(20) $$\begin{array}{c} P_{d}=\int_{0}^{R}P_{u}^{d}f\left(r_{u}\right)dr_{u}=\int_{0}^{R}\left(1-\sum_{i=0}^{L-1}\frac{{\left(\lambda_{u}^{d}T_{d}\right)}^{i}}{i!}e^{-\lambda_{u}^{d}T_{d}}\right)\frac{2r_{u}}{R^{2}}dr_{u}. \end{array}$$

### 3.2. Average Outage Performance of Two-Stage Cooperative Transmission

Similar to Equation (19), after getting the probability density function of $\gamma_{u}^{s1}$, the conditional outage probability of user *u* in stage one can be expressed as

(21) $$\begin{array}{cc} P_{u}^{s1} & =\mathrm{Pr}\left(\gamma_{u}^{s1}<2I_{f}|r_{u}\right)=1-\mathrm{Pr}\left(\gamma_{u}^{s1}\geq T_{s}|r_{u}\right)=1-\sum_{i=0}^{L-1}\frac{{\left(\lambda_{u}^{s1}T_{s}\right)}^{i}}{i!}e^{-\lambda_{u}^{s1}T_{s}}, \end{array}$$

where $T_{s}=2^{2I_{f}}-1$. Accordingly, the conditional outage probability of relay *n* on each codebook is

(22) $$\begin{array}{cc} P_{n}^{s1} & =\mathrm{Pr}\left(\gamma_{n}^{s1}<2^{2I_{f}}-1|r_{n}\right)=1-\mathrm{Pr}\left(\gamma_{n}^{s1}\geq T_{s}|r_{n}\right)=1-\sum_{i=0}^{L-1}\frac{{\left(\lambda_{n}^{s1}T_{s}\right)}^{i}}{i!}e^{-\lambda_{n}^{s1}T_{s}}. \end{array}$$

The probability that relays in N decode the message of any codebook correctly in the first stage is given by

(23) $$\begin{array}{cc} P_{N} & =\prod_{n_{i}\in N}\left(1-P_{n}^{s1}\right)\prod_{n_{i}\notin N}P_{n}^{s1}={\left(\sum_{i=0}^{L-1}\frac{{\left(\lambda_{n}T_{s}\right)}^{i}}{i!}e^{-\lambda_{n}T_{s}}\right)}^{N}{\left(1-\sum_{i=0}^{L-1}\frac{{\left(\lambda_{n}T_{s}\right)}^{i}}{i!}e^{-\lambda_{n}T_{s}}\right)}^{N_{s}-N}. \end{array}$$

In the second stage, when N≥1, the conditional outage probability of user *u* is

(24) $$\begin{array}{c} P_{u}^{s2^{†}}=\mathrm{Pr}\left(\gamma_{{}_{u}}^{s2^{†}}<2I_{f}|r_{u},\theta_{u},N\right)=1-\mathrm{Pr}\left(\gamma_{{}_{u}}^{s2^{†}}\geq 2^{2I_{f}}-1|r_{u},\theta_{u},N\right)=1-\sum_{i=0}^{L-1}\frac{{\left(\lambda_{u}^{s2^{†}}T_{s}\right)}^{i}}{i!}e^{-\lambda_{u}^{s2^{†}}T_{s}}. \end{array}$$

For the case of N=0, the conditional outage probability of user *u* is given by

(25) $$\begin{array}{cc} P_{u}^{s2^{*}} & =\mathrm{Pr}\left(\gamma_{{}_{u}}^{s2^{*}}<2I_{f}|r_{u}\right)=1-\mathrm{Pr}\left(\gamma_{{}_{u}}^{s2^{*}}\geq 2^{2I_{f}}-1|r_{u}\right)=1-\sum_{i=0}^{L-1}\frac{{\left(\lambda_{u}^{s2^{*}}T_{s}\right)}^{i}}{i!}e^{-\lambda_{u}^{s2^{*}}T_{s}}. \end{array}$$

Considering Equations (24) and (25) together, the conditional outage probability of user *u* in stage two can be expressed as

(26) $$\begin{array}{c} P_{u}^{s2}=\left\{\begin{array}{c} P_{u}^{s2^{†}},N\geq 1,\\ P_{u}^{s2^{*}},N=0. \end{array}\right. \end{array}$$

Assuming all Rayleigh channel gains {h} are i.i.d, based on Equations (21), (23), and (26), the average outage probability of *U* users can be expressed as

(27) $$\begin{array}{ccc} P_{s} & = & \sum_{N=0}^{N_{s}}\sum_{|N|=N}\int_{0}^{R}\int_{0}^{2\pi }P_{u}^{s1}\times P_{u}^{s2}\times P_{N}\times f\left(r_{u},\theta_{u}\right)d\theta_{u}dr_{u}\\ & = & \sum_{N=1}^{N}\sum_{|N|=N}{\left(1-P_{n}^{s1}\right)}^{N}\times {\left(P_{n}^{s1}\right)}^{N_{s}-N}\int_{0}^{R}\int_{0}^{2\pi }P_{u}^{s1}\times P_{u}^{s2^{†}}\times f\left(r_{u},\theta_{u}\right)d\theta_{u}dr_{u}\\ & + & \int_{0}^{R}{\left(P_{n}^{s1}\right)}^{N}\times P_{u}^{s1}\times P_{u}^{s2^{*}}\times f\left(r_{u}\right)dr_{u}. \end{array}$$

## 4. Optimal Design of Cooperative Transmission

In this section, the optimal power allocation and relay position $r_{n}$ of cooperative transmission scheme are analyzed. Letting $\alpha =P_{s1}\xi /\left(2P\right)$ be the power allocation factor and $\mu =r_{n}/R$ be the relay location factor, Equation (27) is a function of the average outage probability with variables α and μ. However, it is difficult to derive the optimal α and μ due to the complicated expression of Equation (27). Instead, the approximate value of $P_{s}$ in a high SNR scenario is considered. To facilitate the following analysis, we take C=6, K=4, and L=2 as an example, which is easy to extend to other situations. When $P_{s1}/N_{0}$ is large, Equation (21) can be approximated as

(28) $$\begin{array}{ccc} P_{u}^{s1} & = & 1-e^{-\lambda_{u}^{s1}T_{s}}\left(1+\lambda_{u}^{s1}T_{s}\right)\approx 1-\left(1-\lambda_{u}^{s1}T+\frac{{\left(\lambda_{u}^{s1}T_{s}\right)}^{2}}{2}\right)\left(1+\lambda_{u}^{s1}T_{s}\right)\\ & = & \frac{1}{2}\left({\left(\lambda_{u}^{s1}T_{s}\right)}^{2}-{\left(\lambda_{u}^{s1}T_{s}\right)}^{3}\right)\approx \frac{1}{2}{\left(\lambda_{u}^{s1}T_{s}\right)}^{2}. \end{array}$$

In Equation (28), we use the Taylor series approximation $e^{x}\approx 1+\frac{1}{1!}x+\frac{1}{2!}x^{2}$ for *x* close to 0, which is tight in a high SNR scenario. In addition, the high-order term ${\left(\lambda_{u}^{s1}T_{s}\right)}^{3}$ in Equation (28) is omitted. Equations (22), (24), and (25) can also be approximated as

(29) $$\begin{array}{c} P_{n}^{s1}\approx \frac{1}{2}{\left(\lambda_{n}^{s1}T_{s}\right)}^{2}, \end{array}$$

(30) $$\begin{array}{c} P_{u}^{s2^{†}}\approx \frac{1}{2}{\left(\lambda_{u}^{s2^{†}}T_{s}\right)}^{2}, \end{array}$$

and

(31) $$\begin{array}{c} P_{u}^{s2^{*}}\approx \frac{1}{2}{\left(\lambda_{u}^{s2^{*}}T_{s}\right)}^{2}, \end{array}$$

respectively. Based on Equations (28)–(31), a high SNR approximation of $P_{s}$ can be expressed as Equation (32):

(32) $$\begin{array}{ccc} \tilde{P_{s}}\; & = & \sum_{N=1}^{N_{s}}\sum_{|N|=N}{\left(1-\frac{1}{2}{\left(\lambda_{n}^{s1}T_{s}\right)}^{2}\right)}^{N}{\left(\frac{1}{2}{\left(\lambda_{n}^{s1}T_{s}\right)}^{2}\right)}^{N_{s}-N}\int_{0}^{R}\int_{0}^{2\pi }\frac{1}{2}{\left(\lambda_{u}^{s1}T_{s}\right)}^{2}\frac{1}{2}{\left(\lambda_{u}^{s2^{†}}T_{s}\right)}^{2}\frac{r_{u}}{\pi R^{2}}d\theta_{u}dr_{u}\\ & + & {\left(\frac{1}{2}{\left(\lambda_{n}^{s1}T_{s}\right)}^{2}\right)}^{N_{s}}\int_{0}^{R}\frac{1}{2}{\left(\lambda_{u}^{s1}T_{s}\right)}^{2}\frac{1}{2}{\left(\lambda_{u}^{s2^{*}}T_{s}\right)}^{2}\frac{2r_{u}}{R^{2}}dr_{u}\\ & = & \sum_{N=1}^{N_{s}}\sum_{|N|=N}{\left(1-\frac{1}{2}{\left(\lambda_{n}^{s1}T_{s}\right)}^{2}\right)}^{N}{\left(\frac{1}{2}{\left(\lambda_{n}^{s1}T_{s}\right)}^{2}\right)}^{N_{s}-N}\frac{{\left(T_{s}N_{0}\right)}^{4}}{4\pi {\left(P_{s1}^{}P_{s2^{†}}^{}R\right)}^{2}}\\ & \times & \int_{0}^{R}\int_{0}^{2\pi }{r_{u}}^{2\eta +1}{\left(\sum_{n_{i}\in N}{r_{u,n_{i}}}^{-\eta }\right)}^{-2}d\theta_{u}dr_{u}{\left(\frac{1}{2}{\left(\lambda_{n}^{s1}T_{s}\right)}^{2}\right)}^{N_{s}}\frac{{\left(T_{s}N_{0}R^{\eta }\right)}^{4}}{4{\left(P_{s1}^{}P_{s2^{*}}^{}\right)}^{2}\left(2\eta +1\right)}. \end{array}$$

In Equation (32), the lowest order is $N=N_{s}$, after omitting the terms of $N<N_{s}$ and using the approximation ${\left(\frac{1}{2}{\lambda_{n}}^{2}{T_{s}}^{2}\right)}^{N_{s}}\to 0$, we get the approximation of $\tilde{P_{s}}$:

(33) $$\begin{array}{c} \tilde{P_{s}}\;\approx \frac{{\left(T_{s}N_{0}\right)}^{4}}{4{\left(P_{s1}^{}P_{s2^{†}}\right)}^{2}}\times \underset{\Lambda }{\underset{︸}{\int_{0}^{R}\int_{0}^{2\pi }\frac{{r_{u}}^{2\eta +1}}{\pi R^{2}}{\left(\sum_{n_{i}\in N}{r_{u,n_{i}}}^{-\eta }\right)}^{-2}d\theta_{u}dr_{u}}}. \end{array}$$

### 4.1. Optimal Power Allocation

Based on the approximation of average outage probability, we calculate the optimal power allocation in this section. In Equation (33), Λ depends on the total number of relays $N_{s}$ and their locations $r_{n}$ but not the transmission power. Thus, the problem of power allocation can be expressed as

(34) $$\begin{array}{cc} \min\frac{{\left(T_{s}N_{0}\right)}^{4}}{4{\left(P_{s1}P_{s2^{†}}\right)}^{2}} & =\max P_{s1}P_{s2^{†}}, \end{array}$$

subject to

(35) $$\begin{array}{cc} PT & =\frac{T}{2}\xi \left(P_{s1}+NP_{s2^{†}}\right). \end{array}$$

Since $P_{s2^{†}}$ is the power of all successful relays on each codeword, we need to get the average number of successful relays, which can be obtained by

(36) $$\begin{array}{c} \bar{N}=N_{s}P_{n}^{s1}=N_{s}\left(1-\frac{1}{2}{\left(\lambda_{n}^{s1}T_{s}\right)}^{2}\right). \end{array}$$

In addition, with large $P_{s1}/N_{0}$, $\frac{1}{2}{\left(\lambda_{n}^{s1}T_{s}\right)}^{2}\ll 1$ and we can have $\bar{N}\approx N_{s}$. Replace *N* in Equation (35) with $\bar{N}$, the average value of $P_{s2^{†}}$ can be expressed as

(37) $$\begin{array}{c} \bar{P_{s2^{†}}}\;=\frac{2P-P_{s1}\xi }{\xi N_{s}\left(1-\frac{1}{2}{\left(\lambda_{n}^{s1}T_{s}\right)}^{2}\right)}\approx \frac{2P-\xi P_{s1}}{\xi N_{s}}. \end{array}$$

Therefore, we have

(38) $$\begin{array}{c} P_{s1}P_{s2^{†}}\approx \frac{P_{s1}}{\xi N_{s}}\left(2P-\xi P_{s1}\right), \end{array}$$

and

(39) $$\begin{array}{c} \frac{\partial \frac{P_{s1}}{\xi N_{s}}\left(2P-\xi P_{s1}\right)}{\partial P_{s1}}=0\;\Rightarrow \frac{2(P-\xi P_{s1})}{\xi N_{s}}=0. \end{array}$$

From Equations (38) and (39), we can get the maximum value of $P_{s1}P_{s2^{†}}$ when $P_{s1}\approx P/\xi$. Thus, the optimal power allocation factor is $\alpha =P_{s1}\xi /\left(2P\right)\approx 0.5$, and the average power of each codeword on each relay is $\bar{P_{s2^{†}}}\approx P/\left(\xi N_{s}\right)$.

### 4.2. Optimal Relay Location

Based on the analysis of the optimized power allocation, we investigate the optimal relay location $r_{n}$ in this section. Substituting $P_{s1}\approx P/\xi$ and $\bar{P_{s2^{†}}}\approx P/\left(\xi N_{s}\right)$ into Equation (33), with some algebraic manipulations, the approximate value of average outage probability can be rewritten as

(40) $$\begin{array}{c} \tilde{P_{s}}\;\approx \frac{{\left(T_{s}^{}N_{0}^{}\right)}^{4}}{4{\left(P_{s1}^{}P_{s2^{†}}^{}\right)}^{2}}\Lambda \approx \frac{{N_{s}}^{2}{\left(T_{s}^{}N_{0}\xi \right)}^{4}}{4P^{4}}\Lambda . \end{array}$$

Therefore, the problem of minimizing $\tilde{P_{s}}$ can be approximated to minimize Λ. The expression of Λ can be rewritten as Equation (41), which is a function of μ and $N_{s}$. For Equation (41), we obtain the optimal μ for each $N_{s}$ by Monte Carlo and numerical methods. Table 1 lists the optimal μ with a different number of relays ($N_{s}$ from 2 to 6):

(41) $$\begin{array}{c} \Lambda =\int_{0}^{R}\int_{0}^{2\pi }\frac{{r_{u}}^{2\eta +1}}{\pi R^{2}}{\left(\sum_{n_{i}\in N_{s}}{\left({r_{u}}^{2}+\mu^{2}R^{2}-2r_{u}\mu R\cos\left(\theta_{u}-\frac{(n_{i}-1)2\pi }{N_{s}}\right)\right)}^{-\eta }\right)}^{-2}d\theta_{u}dr_{u}. \end{array}$$

To summarize, the relay design scheme in the high SNR region has been given, and the pseudocode is given by Algorithm 2. In the proposed scheme of relay design, the computational complexity is mainly dominated by the numerical solution of Equation (41), the complexity of which is $O(\frac{1}{t})$ (t=0.01 is the calculation accuracy in this work). In the low SNR region, the computational complexity of the relay design is higher compared to the high SNR region because it is difficult to deal with Equation (27). For the numerical solutions of minimize $P_{s}$ in the low SNR region, its computational complexity is $O(\frac{1}{t^{2}})$.

**Algorithm 2** Optimal design of cooperative transmission.**Input:** transmit power *P*, transmit rate $I_{f}$, number of relays $N_{s}$. **Output:** power allocation factor α, relay location factor μ. 1:According to Equation (33), we can observe that α and μ are two parts that do not affect each other. Then, we can obtain the optimal values of α and μ separately.2:**The calculation of the optimal value of α:**3:According to Equation (34), α is optimal when $P_{s1}P_{s2^{†}}$ reaches the maximum value.4:After obtaining $\bar{N}$ (the average number of successful relays), get the expression of the average value of $P_{s2^{†}}$.5:Establish the equation $P_{s1}P_{s2^{†}}=0$.6:Solving the derivative of the above equation, we have $P_{s1}\approx P/\xi$.7:The optimal power allocation factor is $\alpha =P_{s1}\xi /\left(2P\right)\approx 0.5$.8:**The calculation of the optimal value of μ:**9:Initialization t=0.01, 0≤μ≤1, $N_{s}=2,3,4,5,6$.10:**for**$N_{s}=2:1:6$**do**11: **for** μ=0:t:1 **do**12:  Calculate Λ according to Equation (41).13: **end for**14: Search for the value of μ when Λ is the minimum, and this μ is optimal.15:**end for**16:The calculation is completed.

## 5. Numerical Results and Discussion

In this section, the Monte Carlo simulation and Numerical results based on Matlab are presented to evaluate the performance of the SCMA-based hybrid unicast-multicast network. We assume that the number of OFDMA tones is K=4, the number of non-zero elements in each codebook is L=2, the number of codebooks is C=6 [19,20,21], the target rate is $I_{f}=1$ bit/s/Hz, the radius of the cell is R=100 m, and the path-loss exponent is η=2.6. The OMA is used to compare the performance with SCMA on the basis of same parameters as above. Different from SCMA, the resource unit of OMA is OFDMA tone. For fair comparison, we consider equivalent physical resources in both networks. Thus, the total number of unicast users/multicast groups can be accessed in OMA network is *K*, and the transmit power of each user is *P*. Firstly, the simulation results of the outage probability for different schemes are presented. Then, the accurate and approximate results of average outage probability in the high SNR regime are verified. Finally, the simulation results with optimal relay location and power allocation are demonstrated. $P/\xi N_{0}$ represents the transmit SNR of each codeword in the direct transmission scheme. Accordingly, the transmit SNRs of each codeword in stage one, stage two with N=0 and N≥1 are $2\alpha P/(\xi N_{0})$, $2P\left(1-\alpha \right)/\left(\xi N_{0}\right)$ and $2P\left(1-\alpha \right)/\left(\xi NN_{0}\right)$, respectively.

**Figure 3:**
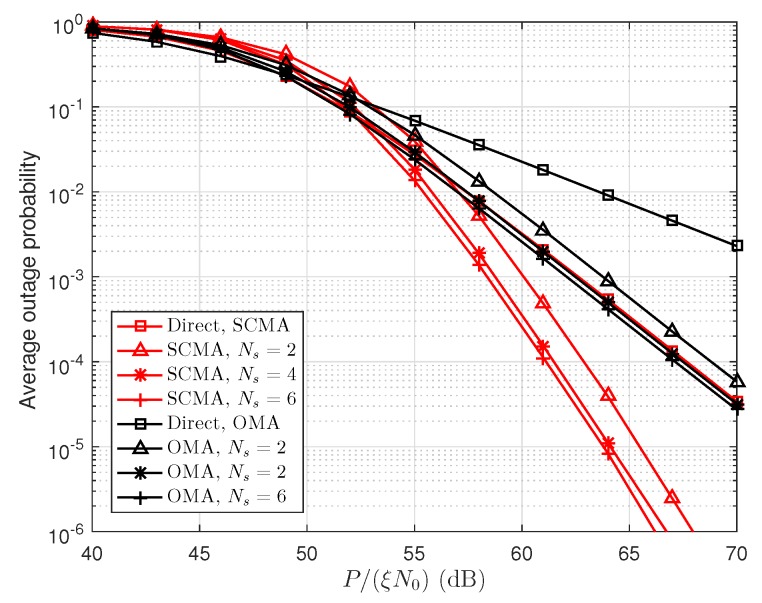
The average outage probability versus $P/(\xi N_{0})$.

We use $N_{s}=2,4,6$ (number of relays), α=0.5 (power power allocation factor) and μ=0.6 (relay location factor) as an example. Figure 3 shows the average outage probability versus $P/(\xi N_{0})$. In Figure 3, the performance of direct transmission and cooperative transmission with different numbers of relays in both SCMA and OMA networks are simulated. We can see that SCMA outperforms OMA in terms of outage probability for both direct and cooperative transmission schemes, which is because users in the SCMA network obtain spreading gain compared to OMA network. The cooperative transmission scheme has lower outage probability compared to direct transmission scheme in the high SNR regime, and the increasing number of relays can reduce the user’s outage probability. For example, maximum gain (about 3 dB) is obtained when $N_{s}$ is increases from 2 to 6. This phenomenon can be explained the fact that the cooperative transmission provides array gain compared to the direct transmission.

**Figure 4:**
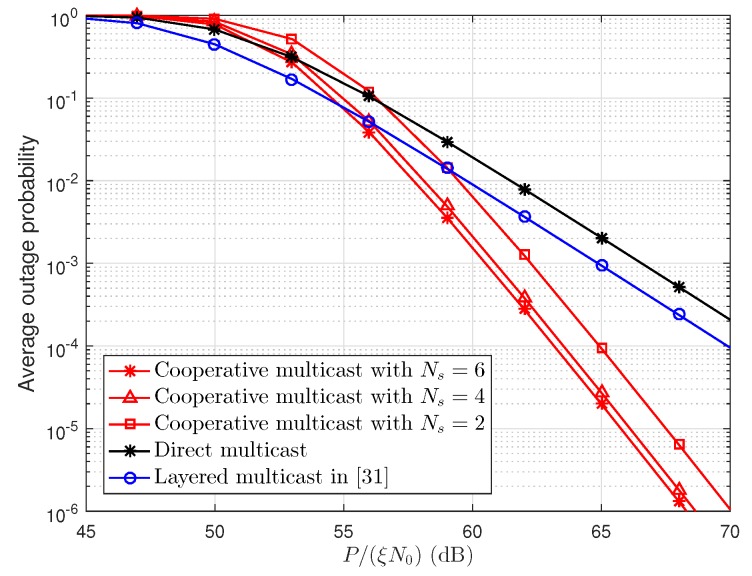
Average outage probability of proposed scheme and scheme in [31].

Currently, there are fewer works focusing on the development of SCMA-based system. In [31], layered multicast is introduced to maximize the multicast system capacity. Although Scheme [31] is different from the target of this work, it would be interesting to compare the outage probability between cooperative multicast and layered multicast. Figure 4 demonstrates the average outage probability of direct multicast, cooperative multicast with $N_{s}=2,4,6$, and layered multicast in the SCMA network. In Figure 4, all *C* codebooks are assigned to one multicast group, and other parameters are the same as in Figure 3. It can be seen that the layered multicast has a better outage performance than other schemes in low SNR regime. This is because the users has higher outage probability when the SNR is lower. Moreover, to guarantee the minimum service quality of all users, the layered multicast allocates more resources to the basic layer. As the transmit SNR increases, cooperative multicast obtains an array gain. Therefore, cooperative multicast outperforms layered multicast [31] in terms of outage probability in the high SNR regime.

**Figure 5:**
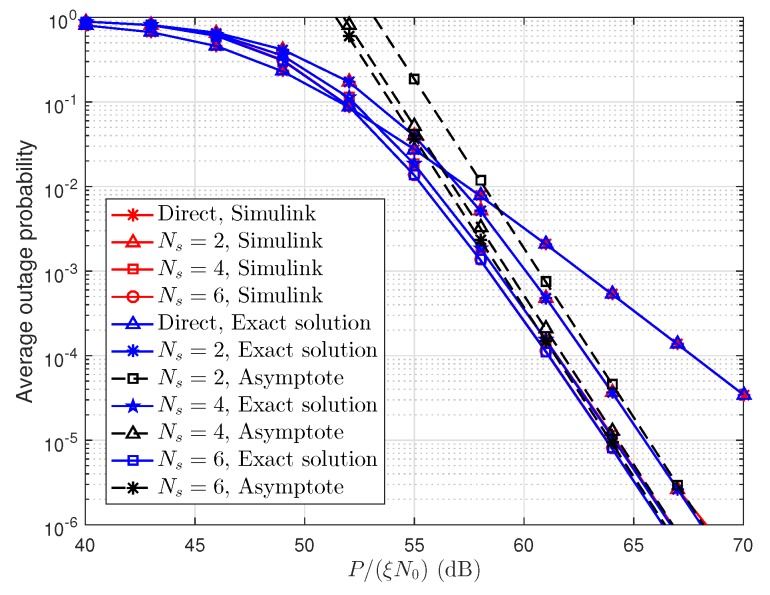
Comparison of simulation, exact and approximated average outage probabilities.

In Figure 5, the simulation and exact (Equation (20)) results of the direct transmission scheme are presented. Meanwhile, the simulation, exact (Equation (27)), and approximate (Equation (33)) results of the two-stage cooperative transmission scheme are also presented. The simulation parameters are the same as in Figure 3. We can clearly see that the exact outage probability curves are in good agreement with the Monte Carlo simulation results. The asymptotic curves almost overlap with the exact performance curves when $P/(\xi N_{0})$ is large, which verifies our derivation of the average outage probability in the high SNR regime.

**Figure 6:**
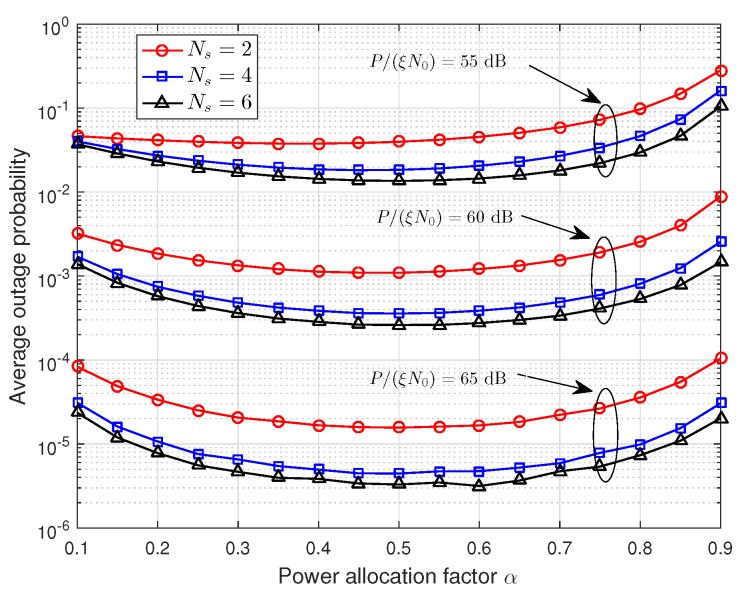
The effect of power allocation factor α on the average outage probability with different numbers of relays.

**Figure 7:**
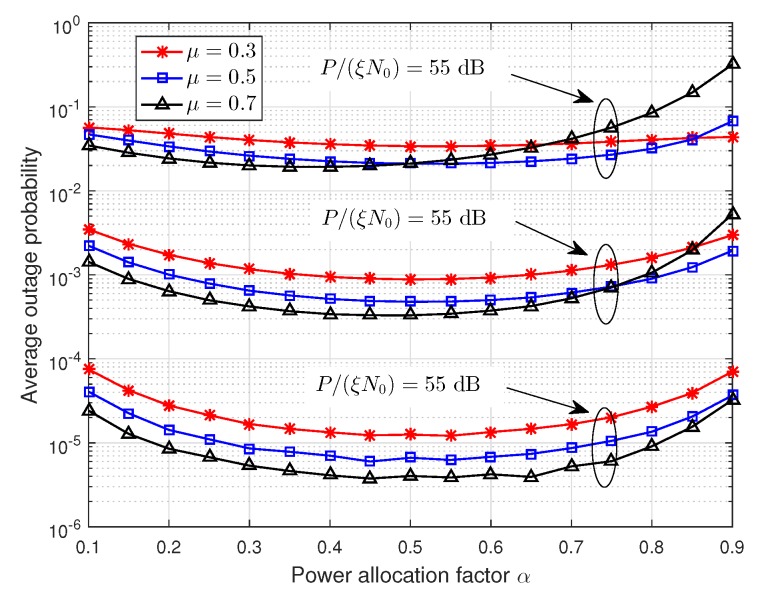
The effect of power allocation factor α on the average outage probability with different relay locations.

Figure 6 and Figure 7 show that the effect of power allocation factor α on the average outage probability, where $P/(\xi N_{0})=55,60,65$ dB are used as examples. In Figure 6, the number of relays is $N_{s}=2,4,6$, and μ=0.6. In Figure 7, the number of relays is $N_{s}=4$, and μ=0.3,0.5,0.7. From these two figures, we can observe that the power allocation factor has a significant impact on the average outage probability. Thus, it is necessary to find out the optimal power allocation factor. Additionally, the average outage probability decreases first and then increases with the increasing of power allocation factor α, and it reaches the minimum value when α≈0.5. This is consistent with the conclusion in Section 4.1, i.e., the first stage occupies half of the total energy consumed.

**Figure 8:**
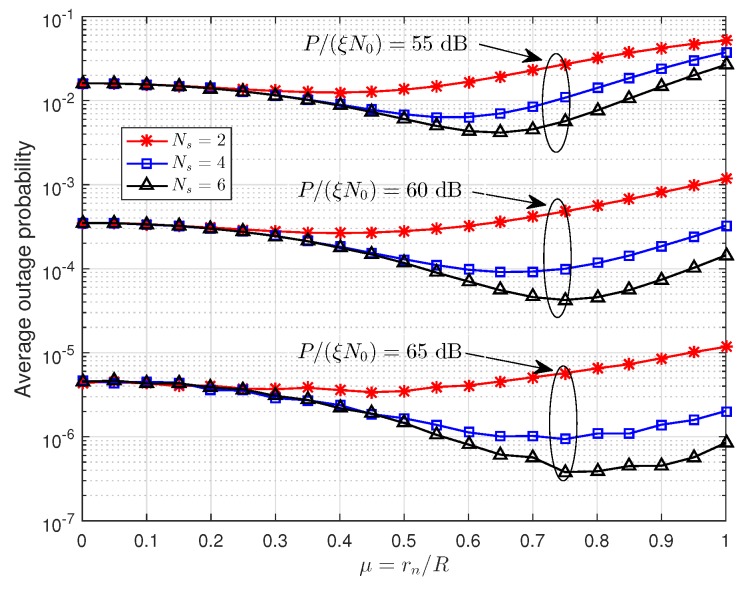
The effect of relay location on the average outage probability.

Figure 8 gives the trend of outage probability with the locations of relays, in which optimal power allocation factor α=0.5 is used. From this figure, we can observe that the μ in Table 1 achieve the lowest outage probability when $P/(\xi N_{0})$ is greater than 60 dB, which verifies our results from Section 4.2. It is worth noting that the results of Table 1 are obtained in high SNR region and the optimal power allocation and relay positions in the low SNR region (e.g., when $P/(\xi N_{0})=55$ dB) may be different from our analysis in Section 4.

**Figure 9:**
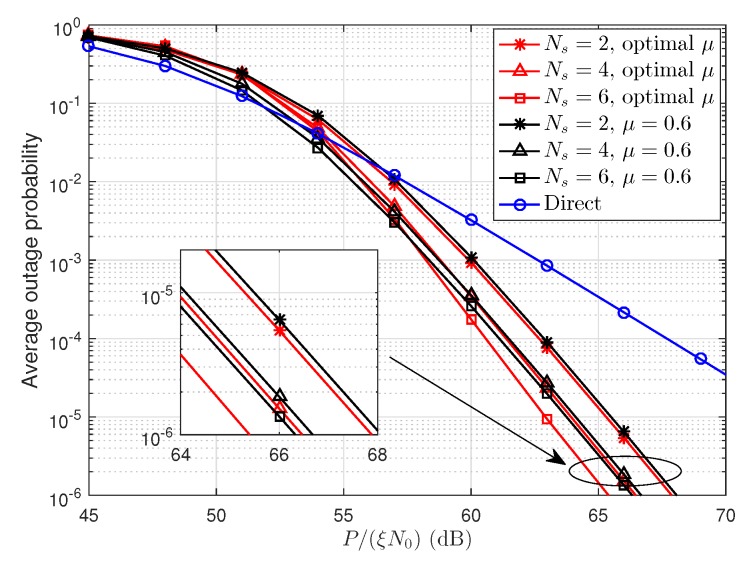
The average outage probability versus $P/(\xi N_{0})$ with optimal power allocation and optimal relay location.

Using optimal power allocation factor α=0.5 and optimal relay locations specified in Table 1, Figure 9 shows the average outage probability versus $P/(\xi N_{0})$. We can see that optimal relay design effectively reduce the average outage probability. For example, the maximum gain (about 1 dB) is obtained when $N_{s}=6$. The cooperative transmission scheme achieves a maximum gain of 8 dB compared to the direct transmission scheme.

**Figure 10:**
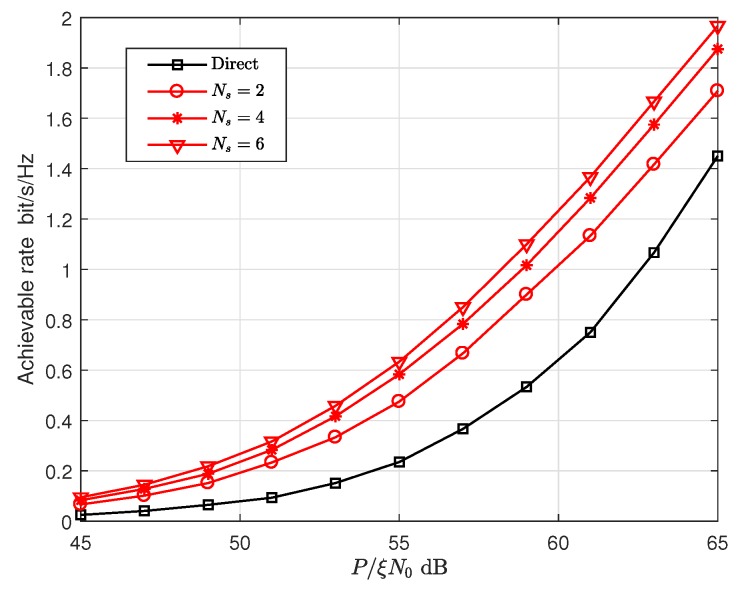
The achievable rate versus $P/(\xi N_{0})$ with optimal power allocation and optimal relay location.

**Figure 11:**
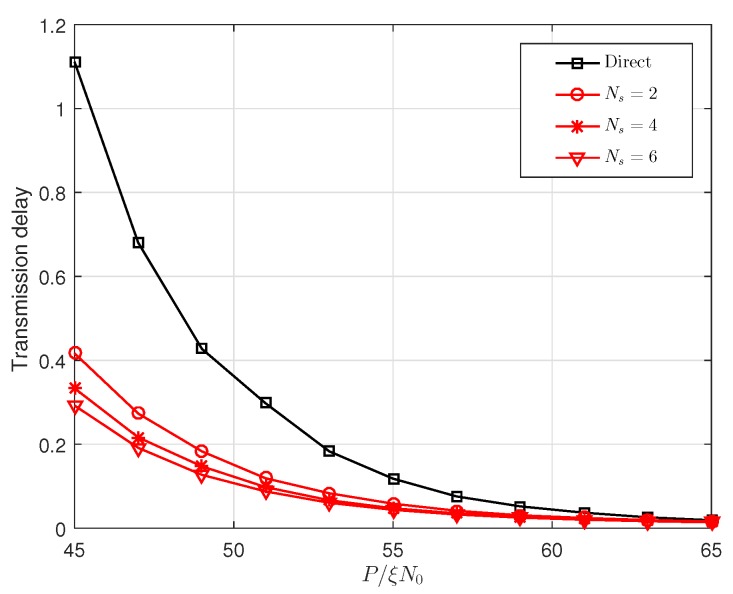
The transmission delay versus $P/(\xi N_{0})$ with optimal power allocation and optimal relay location.

Figure 10 and Figure 11 show that the effect of $P/(\xi N_{0})$ on the achievable rate and the transmission delay, respectively, where the optimal power allocation factor and optimal relay locations are used. The achievable rate is the maximum allowable transmission rate of each codebook under the constraint of the outage probability ($10^{-3}$ in Figure 10 and Figure 11). The delay is the time required to transmit a packet at the achievable rate. To be more intuitive, we assume that the size of the packet is 1, and then the delays in Figure 11 are the time required to transmit a unit packet. From the two figures mentioned, we can see that the proposed cooperative transmission has a higher achievable rate and lower delay than the direct transmission scheme.

## 6. Conclusions

In this paper, the application of SCMA to a multi-user hybrid unicast-multicast network is considered, and the two-stage cooperative transmission scheme is introduced to reduce the average outage probability. Firstly, the mathematical characteristics of outage probability in direct transmission and cooperative transmission schemes are analyzed. Based on these analyses, the expressions of the average outage probability in both schemes are derived. Secondly, to further improve the performance of cooperative transmission scheme, the relay design is considered. Accordingly, optimal relay location and power allocation factor are given by mathematical analysis. Finally, both analytical and simulation results verify that the cooperative transmission has a significant improvement in outage performance compared to direct transmission. The results indicate that the outage performance of SCMA surpasses OMA in these two transmission schemes. Specifically, the analysis results can be applied to any number of multicast groups (e.g., all codebooks are allocated to multicast groups or unicast users) in SCMA networks.

## Figures and Tables

**Table 1:** Asymptotic optimal relay locations.

Number of Relays $N_{s}$	2	3	4	5	6
$\mu =r_{n}/R$	0.405	0.635	0.741	0.790	0.821
